# Personal and contextual variables predict music consumption during the first COVID-19 lockdown in Canada

**DOI:** 10.3389/fpsyg.2023.1116857

**Published:** 2023-06-14

**Authors:** Yuvika Dandiwal, Lindsay Fleming, Daniel J. Levitin

**Affiliations:** Department of Psychology, McGill University, Montreal, QC, Canada

**Keywords:** music listening, COVID-19, worry, music watching, music discovery, coping, individual differences

## Abstract

The global COVID-19 lockdowns shattered familiar routines, plunging individuals into a disorienting emotional landscape characterized by loss, uncertainty, and a deep yearning for social bonds. Many employed coping strategies such as cleaning, dancing, and mindfulness-based practices to ameliorate negative emotions. Music listening was one of the most widely used coping strategies, moderated by personal and contextual variables. We obtained data from a Canadian national survey conducted in April 2020 to examine the role of personal (sex, age, education level, pre-pandemic income, minority status, feelings about music, and Schwartz’s “personal values”) and contextual variables (level of worry, changes to income, COVID-19 status and risk, having children at home, and internet access) in predicting music listening for stress relief, changes to music listening, changes to music watching, and music discovery. Our results indicate that women, younger adults, individuals who like or love music, and those reporting high levels of worry were more likely to listen to music to relieve stress. Personal variables were more significantly associated with music listening for stress relief than contextual variables.

## Introduction

The COVID-19 pandemic caused catastrophic economic and social disruption, claimed millions of lives, posed unprecedented challenges to public health, and presented an existential threat to millions of individuals and businesses across the globe. In Canada, COVID-19 was first identified in January 2020 and spread nationwide by March. The virus had far-reaching physical, economic, social, and political consequences, and its profound psychological impact in particular is noted in the scientific literature (Alkhamees et al., 2020; Brooks et al., 2020; Hossain et al., 2020; Rodríguez-Rey et al., 2020; Serafini et al., 2020; Passavanti et al., 2021). For those directly affected by COVID, the psychological impact includes increased prevalence of depression, anxiety, sleep disorders, and low self-esteem due to physical isolation, stay-at-home orders, and government-imposed lockdowns (Hossain et al., 2020). Even among those indirectly affected, long-lasting posttraumatic stress symptoms have been observed, due to the fear of infection, financial loss, and shortages in food and basic household necessities (Brooks et al., 2020).

To manage increasing mental health challenges and emotional distress, individuals employed various coping strategies and engaged with numerous activities. A wide range of strategies such as watching television, social networking, listening to music, and sleeping were employed (Sameer et al., 2020). Music listening was used extensively (Fink et al., 2021; Finnerty et al., 2021). Engaging in leisure activities such as music, as well as home crafts and artisanship, fine arts, performing arts, sports, and outdoor pursuits correlated highly with well-being among participants across 74 countries (Morse et al., 2021). In a cross-cultural survey across 11 countries, music was more or as effective as other strategies (hobbies, physical activity, information-seeking, reading, eating/cooking, doing productive activities, mindfulness, movie watching) in meeting wellbeing goals (Granot et al., 2021). In the midst of the pandemic, Greenberg et al. (2021) observed a remarkable capacity for adaptation in many individuals, who swiftly adjusted to the restrictions on social interaction and isolation, finding new ways to meet their social needs.

We humans are a social species, and music has been used to facilitate social interaction for tens of thousands of years (Mithen, 2006; Conard et al., 2009). It taps into our intrinsic need for social connection, for, e.g., by providing comfort through emotional contagion among listeners and performers (Egermann and McAdams, 2013). Music can also boost the immune system, reduce stress, and improve physical and mental health and well-being across the lifespan (Chanda and Levitin, 2013; Levitin, 2019; Sheppard and Broughton, 2020). The potential of music listening for improving well-being and alleviating mental distress is demonstrated by the adoption of in-person and virtual music interventions during the pandemic to reduce feelings of anxiety or worry among different clinical populations (Giordano et al., 2022; Vinciguerra and Federico, 2022).

The reciprocal-feedback model (Hargreaves et al., 2005; Hargreaves, 2012) proposes that personal, contextual, and musical factors interact with each other in complex ways to influence music learning and engagement. Drawing on this model, we explore how these factors may be relevant to the efficacy of music for socio-emotional coping. For example, differences in personality traits (Chamorro-Premuzic et al., 2009) and gender (Livesey et al., 2012) affect these uses, with women more likely than men to listen to music for mood regulation and stabilization (Lonsdale and North, 2011; Chamorro-Premuzic et al., 2012). The experience sampling method (ESM), which assesses current subjective experience during natural everyday music episodes, adds a situational perspective: individual differences are of less importance, with situational variables (e.g., listening alone vs. with others, time of the day, self-chosen music, and initial mood state) better predicting both function of music listening and music selection (Randall and Rickard, 2017; Greb et al., 2019).

An emerging body of research over the last 2 years has explored (changes in) music listening, the role that music has played in people’s lives during the pandemic, and the personal and contextual variables that predict engagement with music (Hansen et al., 2022) (unfortunately, studies do not ask the same questions, limiting direct comparison). In a sample of Israeli residents, amount of music listening during the pandemic remained constant or increased (Ziv and Hollander-Shabtai, 2022), while Spanish citizens reported an increase in the perception of time devoted to music activities such as music listening, singing, dancing, or playing an instrument (Cabedo-Mas et al., 2021). Roese and Merrill (2021) found that individuals in Germany reduced their active engagement with music (e.g., seeking music-related information) while their passive engagement (e.g., music listening to kill time) increased.

Personal variables such as age, gender, and personality traits have been found to influence music use during the pandemic. Younger individuals (aged 18–24) were more likely to integrate music into daily life, use music for emotion regulation, respond to music in embodied ways, and use music to perform a social identity than older adults (Chmiel et al., 2022). After the first COVID-19 case was confirmed in a given country, the sentiment of music accessed on Last.fm became more negative. This negative effect was more pronounced for male users than female users (Liu et al., 2020). Individuals higher in extraversion reported using music differently during the lockdown, reflecting changes in daily life, missing social (music listening) events, and the need to adapt music listening routines (Roese and Merrill, 2021). Contextual variables, such as music listening opportunities and routines, are also predictors; for example, the lack of a regular commute and fitness center closures led to a decrease in music listening for some (Carlson et al., 2021).

Researchers have also sought to identify why people listened to music during the pandemic (Fink et al., 2021), how these reasons might differ from pre-pandemic motivations, and personal and contextual variables that moderate use of music for specific functions. Individuals reported listening to music to relax, to escape, to elevate their mood, or to have some company (Cabedo-Mas et al., 2021; Ferreri et al., 2021). Music was one of the most commonly reported coping strategies for stress among university students during the pandemic (Vidas et al., 2021). Music consumption was a significant mediator between stress and coping efficacy (Nabi et al., 2022) listening to music was associated with lower levels of stress during the pandemic (Feneberg et al., 2023).

Martínez-Castilla et al. (2021) examined personal variables (age, gender, musical training, personality, resilience, and perception of music’s importance) and contextual variables (living in a region with a high COVID-19 impact, perception of belonging to a risk group, being alone, having caring responsibilities during the lockdown, and music listening compared to before the pandemic). They found an effect of personal variables only on music’s perceived efficacy for well-being; importance of music in life was the most significant factor. In line with previous literature, the youngest adults and those with musical training experienced the highest efficacy of music for well-being. They found no significant effect of gender. Granot et al. (2021) also found age to predict the efficacy of music, but only for obtaining Enjoyment or Togetherness; gender did not have an effect on any of the well-being goals. Martín et al. (2021) found that age moderated music listening goals: individuals between 18 and 40 years old listened to music to alleviate loneliness while those over 51 were more likely to listen to music for emotional self-regulation. During the pandemic, personality traits also predicted the ways individuals used music to regulate emotions (Drake et al., 2022). Extraversion and openness to experience predicted the use of the approach and self-development strategies of emotional regulation, while conscientiousness predicted the use of the avoidance strategy.

Personal values have been described as the missing link in creating meaningful social bonds through music (Boer et al., 2011) and a neglected variable in individual differences in music listener motivations (Manolika et al., 2021). Few studies have investigated the relationship between personal values and music use, but they have been shown to impact both musical preferences (Gardikiotis and Baltzis, 2012) and uses of music (Manolika et al., 2021). Schwartz’s Personal Values Classification (PVC) is a theoretical framework that categorizes individual values into 10 distinct values: achievement, power, hedonism, stimulation, self-direction, universalism, benevolence, tradition, conformity, and security (Schwartz, 1992, 2012). Individuals who place a higher importance on self-transcendent values (universalism and benevolence) are more likely to use music for emotional regulation; the higher-order value of openness to change (comprised of stimulation, self-direction, and hedonistic values) correlates with a preference for music highlighting complex and intense emotions (Manolika et al., 2021).

These values are posited as the underlying motivations that serve as guiding principles for attitudes and behavior. They tend to be largely stable over time and across contexts (Schuster et al., 2019) and can be conceived of as traits that help to predict one’s behavior in a sea of changing situations. The rank order stability of PVC values was quite high in one Australian study specific to the COVID-19 pandemic; however, following the pandemic’s onset, a mean increase in conservation (comprised of conformity, tradition, and security) and decrease in openness to change was observed across participants, especially among worried individuals (Daniel et al., 2022). To our knowledge, no studies have investigated the relationship between Schwartz’s Personal Values Classification (PVC) and music use during the COVID-19 pandemic. Various studies have examined the relationship between the Big 5 personality traits and PVC values (Roccas et al., 2002; Haslam et al., 2009; Parks-Leduc et al., 2015) and have found some overlap between the two constructs. For instance, Parks-Leduc et al.’s (2015) meta-analysis of 60 studies found a consistent and theoretical relationship between the two showing that cognitively-based traits, such as openness to new experience, manifested a stronger relationship with values than emotion-based traits. Openness to experience was correlated with PVC values of self-direction, stimulation and universalism. However, while there is some overlap between the Big 5 personality traits and PVC values, the pattern of results in these studies has shown that they are still distinct constructs (Roccas et al., 2002; Parks-Leduc et al., 2015). Our study was restricted to examining PVC values in relation to music use.

Several studies have investigated the role of personal and contextual variables in predicting music listening during the pandemic (e.g., Finnerty et al., 2021; Hennessy et al., 2021; Martínez-Castilla et al., 2021; Roese and Merrill, 2021), no study, to our knowledge, has examined the influence of personal vs. contextual variables on two other forms of music engagement: music watching and music discovery. To address these gaps in literature, we describe an investigation of personal variables (sex, age, feelings about music, minority status, education level, income level, and PVC values) and contextual variables (level of worry, change in income, having children at home, living in a high-risk region, and internet accessibility) in predicting music consumption (that is, discovery of new music and changes to music listening and watching), as well as using music listening for stress relief, among a sample of Canadian adults. Context here refers to the broad context of the COVID-19 pandemic and its initial lockdown, not the specific contexts (i.e., time, place, mood) of music listening. Our findings on the role of personal and contextual variables in predicting music consumption behaviors and functions complement recent findings about the pandemic.

Based on previous reports, we hypothesize that women and younger individuals are more likely to engage in music listening for stress relief. We also predict that demographic variables (sex, age, minority status, education, income) will influence changes to music consumption. We predict individuals who report a strong liking towards music are more likely to engage in music consumption, as well as music listening for stress relief. We further hypothesize that individuals who rate high on Schwartz’s openness to change dimension (corresponding to values of self-direction, stimulation, and hedonism) are more likely to engage in music consumption, and we expect personal values to influence listening to music for stress relief.

In our investigation of contextual variables, we hypothesize that high levels of worry predict increases in music consumption, as well as the use of music for stress relief. We hypothesize that decreased income, a known stressor, predicts an increase in music consumption and the use of music for stress relief. Based on Martínez-Castilla et al. (2021), we hypothesize that neither having children at home nor living in a region with a high incidence of COVID-19 will influence the use of music for stress relief, but will predict increases in music consumption. Finally, we hypothesize home internet accessibility predicts increases in music consumption, due to facilitating availability, and therefore the use of music for stress relief.

In our comparison of personal and contextual variables in relation to music consumption, including listening to music for stress relief, we hypothesize that both personal and contextual variables predict outcomes. We also hypothesize that personal variables better predict music listening for stress relief.

## Materials and methods

Music Canada, an NGO and advocacy organization for the Canadian music industry, commissioned a study by Abacus Data, a Canadian polling and market research firm (2022), who made the data available to us as part of a SSHRC-sponsored effort to better understand the longer-term impacts of the pandemic—and the ensuing economic slowdown—on individuals, businesses and communities. Participants [*N* = 2,500, Male = 45.7%, Female = 54.3%, Mean age = 47.2 years (SD = 16.53)] were invited to respond to the survey on the Lucid Exchange platform. The survey, conducted from April 24 to April 30, 2020, comprised a 168-item questionnaire measuring demographic variables, music engagement before and during the COVID-19 lockdown, risk perception of in-person music and social events in the future, and personal values (Schwartz’s Personal Values Classification or PVC). The sample data closely approximated the Canadian population in terms of provincial population distribution, age distribution, and sex distribution (see Supplementary Table S1 for demographic description of sample). Individual variables relevant to the hypotheses are described below.

### Measures

Independent variables (personal):

1. **Sex, Age, Feelings about music, minority status, education level, and income**: The survey included questions about participants’ demographic information and feelings about music (see Supplementary Table S1).
2. **Values (PVC)**: The respondents were asked to rate how much they identified with 19 different trait statements from the Portrait Values Questionnaire (PVQ) (Schwartz et al., 2001) to measure fundamental personality values and motives. Each statement corresponded to one of the 10 PVC values: Benevolence, Universalism, Security, Achievement, Conformity, Tradition, Hedonism, Self-Direction, Stimulation and Power (see Supplementary Table S2 for explanations of terms). Responses were ranked on a 6-point Likert scale ranging from (1) “Very much like me” to (6) “Not at all like me.” Scores from statements corresponding to each value were combined to obtain an aggregate score.

Independent variables (contextual; see Supplementary Table S3):

1. **Level of worry:** One item in the survey assessed the level of worry experienced by the participants. The answers were measured on a 5-point Likert scale ranging from “Not worried at all” to “Extremely worried.”
2. **Change to income:** One survey item assessed changes to household income since the beginning of the pandemic. The answers were measured on a 5-point Likert scale ranging from “Decreased a lot” to “Increased a lot.”
3. **Children at home:** One binary item in the survey asked if participants have children under the age of 18 in their homes.
4. **Living in a region with high COVID-19 incidence:** Survey items included province of residence, region within the province, and first three characters of postal code. This information was compared to national databases of incidence rates from April 24 to April 30, 2020. According to media reports and official public health data four regions in Quebec (Montreal, Laval, Lanaudière, and Montérégie) and four regions in Ontario (Toronto, the Greater Toronto Area, Ottawa, and the Peel Region) had high COVID-19 case numbers at this time (CBC News, 2020a,b; CTV News, 2020).
5. **Internet speed/how participants access the internet:** One survey item asked if respondents had reliable, high-speed internet access at home. A second item asked whether respondents primarily accessed the internet via a mobile carrier or home internet plan. Internet speed and how participants accessed the internet were considered separate variables in the analyses.
6. **You or someone you knew caught COVID-19:** One survey item asked if respondents or someone they knew personally contracted COVID-19.

Dependent variables (see Supplementary Table S4):

1. **Listening to music for stress relief:** The respondents were asked if listening to music had become a way to relieve stress. The responses were measured on a 4-point Likert scale ranging from “Strongly agree” to “Strongly disagree.”
2. **Change in music listening:** One item in the survey assessed the level of change in listening to music since the start of the COVID-19 pandemic. The answers were measured on a 5-point Likert scale ranging from “Much more than usual” to “Much less than usual.”
3. **Change in music watching:** The respondents were asked about the change in their music watching behaviors during the COVID-19 lockdown. The music watching behaviors assessed were as follows: “Watching music videos,” “Watching recorded live concerts” and “Watching online video content from musicians.” The responses were measured on a 5-point Likert scale, namely, “Much more than usual” to “Much less than usual.” These three survey items were combined to form one variable and named “Music watching.”
4. **Music discovery:** The respondents were asked about their music discovery behaviors during the COVID-19 lockdown. The music discovery behaviors assessed were as follows: “Discovering new musicians and artists” and “Finding new content about the music and musicians I love.” The responses were measured on a 5-point Likert scale ranging from “Strongly agree” to “Strongly disagree. These two survey items were combined to form one variable and named “Music discovery.”

## Statistical analyses

Kruskal-Wallis non-parametric ANOVA and Dwass-Steel-Chritchlow-Fligner (DSCF) pairwise comparisons were conducted using Jamovi 2.3 (Version 2.3, Jamovi project, 2022) to examine the relationship between each dependent variable (music listening for stress relief, changes in music consumption) and each of the personal and contextual variables. Hierarchical linear regression was conducted using IBM SPSS 27.0 (Version 27, IBM Corp, 2017) to test the effect of personal and contextual variables on music listening for stress relief, changes to music listening, changes to music watching, and music discovery with four model blocks. Herein, the first model comprised personal variables of age, sex, feelings about music and the PVC values. The second model comprised the model 1 variables and context related variables of level of worry, change in income, you or someone you knew caught COVID-19, children at home, and living in a region of high incidence of COVID-19. Model 3 comprised Model 1, 2 and additional personal variables of education level, visible minority status, and income level, as well as the context variables of high-speed internet access and type of internet plan. Model 1 and 2 comprised previously known or examined predictors of music consumption, while Model 3 comprised additional variables that we hypothesized may impact music listening for stress relief, changes in music listening, changes in music watching, and music.

## Results

### Analyses

#### Personal variables predicting music listening to relieve stress

About 25% of the participants “Strongly Agreed” (*n* = 615) and 54% “Agreed” (*n* = 1,356) listening to music had become a way of relieving stress over the few weeks prior to the survey. Results of a Kruskal-Wallis ANOVA showed that women were more likely than men to listen to music to relieve stress (*χ*^2^ = 7.75, *p* < 0.01, *ε*^2^ = 0.003). A significant difference was also observed for members of different age groups (*χ*^2^ = 81.9, *p* < 0.001, *ε*^2^ = 0.03). DSCF pairwise comparison indicated the youngest age group (18–29) was more likely to listen to music for stress relief than those in the 30–44 (*W* = −3.94, *p* < 0.05), 45–59 (*W* = −6.47, *p* < 0.001), and 60 and over (*W* = −12.06, *p* < 0.001) age groups. Respondents aged 60 and over reported lower levels of music listening for stress relief than those aged 30–44 (*W* = −9.11, *p* < 0.001) and 45–59 (*W* = −6.46, *p* < 0.001). There was no significant difference between those in the 30–44 and 45–59 age groups. Respondents who indicated they were members of a visible minority were more likely to listen to music to relieve stress than non-minorities (*χ*^2^ = 4.73, *p* < 0.05, *ε*^2^ = 0.002). There were no significant differences for highest level of education completed or pre-pandemic income.

Listening to music for stress relief was found to differ significantly based on respondents’ feelings about music (*χ*^2^ = 454, *p* < 0.001, *ε*^2^ = 0.18). Respondents who reported loving music were more likely to use music for stress relief than those who like it (*W* = 23.78, *p* < 0.001), dislike it (*W* = 7.21, *p* < 0.001), and were indifferent to it (*W* = 21.50, *p* < 0.001). Those reporting to like music were more likely to use music for stress relief than those who dislike it (*W* = 5.3, *p* < 0.05) and were indifferent to it (*W* = 11.90, *p* < 0.001).

Listening to music for stress relief was found to differ significantly (*p* < 0.001) for each of the 10 PVC values (self-direction, power, stimulation, achievement, tradition, universalism, conformity, security, benevolence, and hedonism). Results are outlined in Table 1. Significant pairwise comparisons are listed in Supplementary Table S5.

**Table 1:** Kruskal-Wallis non-parametric ANOVA for PVC values.

		Music for stress relief	Music listening	Music watching	Music discovery
Self-direction	*χ*^2^	99.08	8.73	5.47	67.34
	*p*	**<0.001**	0.12	0.36	**<0.001**
	*ε*^2^	0.04	0.00	0.00	0.03
Benevolence	*χ*^2^	105.87	5.85	7.56	35.92
	*p*	**<0.001**	0.32	0.18	**<0.001**
	*ε*^2^	0.04	0.00	0.00	0.01
Universalism	*χ*^2^	68.79	7.23	12.58	24.7
	*p*	**<0.001**	0.20	**0.03**	**<0.001**
	*ε*^2^	0.03	0.00	0.01	0.01
Hedonism	*χ*^2^	111.49	7.62	5.79	44.11
	*p*	**<0.001**	0.18	0.33	**<0.001**
	*ε*^2^	0.04	0.003	0.002	0.02
Security	*χ*^2^	78.16	3.2	5.1	13.45
	*p*	**<0.001**	0.67	0.40	**0.02**
	*ε*^2^	0.03	0.00	0.00	0.01
Achievement	*χ*^2^	74.6	16.6	11.9	93.8
	*p*	**<0.001**	**0.01**	**0.04**	**<0.001**
	*ε*^2^	0.03	0.007	0.005	0.038
Conformity	*χ*^2^	26.82	1.74	8.37	16.54
	*p*	**<0.001**	0.88	0.14	**0.01**
	*ε*^2^	0.01	0.00	0.00	0.01
Stimulation	*χ*^2^	119.8	22.4	14.2	145.9
	*p*	**<0.001**	**<0.001**	**0.02**	**<0.001**
	*ε*^2^	0.05	0.01	0.01	0.06
Tradition	*χ*^2^	88.5	4.36	11.22	13.19
	*p*	**<0.001**	0.50	**<0.05**	**0.02**
	*ε*^2^	0.04	0.00	0.00	0.01
Power	*χ*^2^	27.35	5.21	7.44	36.72
	*p*	**<0.001**	0.39	0.19	**<0.001**
	*ε*^2^	0.01	0.00	0.00	0.01

Bold values indicate statistical significance.

#### Contextual variables predicting music listening to relieve stress

A Kruskal-Wallis AVOVA for level of worry and listening to music for stress relief revealed significant differences (*χ*^2^ = 18.5, *p* < 0.001, *ε*^2^ = 0.01). Those who reported being extremely worried were more likely to listen to music for stress relief than those who reported being a little worried (*W* = 5.14, *p* < 0.001) and somewhat worried (*W* = 4.17, *p* < 0.05). No other between-group significant differences were found. Respondents with children under the age of 18 in their home were more likely to listen to music for stress relief (*χ*^2^ = 15.1, *p* < 0.001, *ε*^2^ = 0.01). Income change was a significant predictor of music listening for stress relief (*χ*^2^ = 15.1, *p* < 0.01, *ε*^2^ = 0.01). Respondents whose income decreased a lot (1 on a 5-point Likert scale) were more likely than those whose income was unaffected to listen to music to relieve stress (*W* = −5.29, *p* < 0.01). No other between-group significant differences were found for change in income. Living in a region with a high COVID-19 incidence rate was not a significant predictor. Respondents who had COVID-19 themselves or knew someone personally who did were also not significantly more likely to listen to music for stress relief. Internet type (mobile vs. home internet) and internet speed were also not significant predictors of music listening for stress relief.

#### Personal variables predicting changes to music listening

Results of a Kruskal-Wallis ANOVA showed that age was a significant predictor of changes to music listening (*χ*^2^ = 21, *p* < 0.01, *ε*^2^ = 0.01). DSCF pairwise comparison indicated the youngest age group (18–29) significantly increased their music listening compared those in the 45–59 (*W* = −3.63, *p* < 0.05) and 60 and over (*W* = −5.29, *p* < 0.01) age groups. Respondents aged 60 and over increased their music listening less than those aged 30 to 44 (*W* = −5.33, *p* < 0.001). There were no other significant differences between groups. Respondents who indicated they were members of a visible minority increased music listening more than non-minorities (*χ*^2^ = 10.7, *p* < 0.01, *ε*^2^ = 0.004). Education level was also a significant predictor of changes to music listening (*χ*^2^ = 23.1, *p* < 0.001, *ε*^2^ = 0.01). DSCF pairwise comparison indicated those with a high school education increased their music listening by less than those with some college or university (*W* = 4.08, *p* < 0.05) and those who obtained Bachelor’s degree (*W* = 6.5, *p* < 0.001). There were no other significant differences between groups. There were also no significant differences for sex or pre-pandemic income.

Changes to music listening were also found to differ significantly based on respondents’ feelings about music (*χ*^2^ = 53.5, *p* < 0.001, *ε*^2^ = 0.02). Respondents who reported loving music increased their music listening more than those who like it (*W* = 6.74, *p* < 0.001) and were indifferent to it (*W* = 8.4, *p* < 0.001). Those reporting to like music increased their music listening more than those who were indifferent to it (*W* = 4.81, *p* < 0.01). There were no other significant differences between groups.

Changes to music listening differed significantly for the basic values of stimulation (*χ*^2^ = 22.4, *p* < 0.001, *ε*^2^ = 0.01) and Achievement (*χ*^2^ = 16.6, *p* < 0.01, *ε*^2^ = 0.01). Participants who responded “Very much like me” to stimulation items reported smaller increases in music listening than those who responded “Not like me” (*W* = −4.93, *p* < 0.01) and “Not like me at all” (*W* = −5.45, *p* < 0.01). Participants who responded “Very much like me” to achievement items reported smaller increases in music listening than those who responded “Not like me” (*W* = −4.19, *p* < 0.05) and “Not like me at all” (*W* = −4.29, *p* < 0.05).

#### Contextual variables predicting changes to music listening

A Kruskal-Wallis AVOVA for level of worry and changes to music listening revealed significant differences (*χ*^2^ = 19.7, *p* < 0.001, *ε*^2^ = 0.01). Participants who reported being extremely worried increased their music listening more than those who reported being not worried (*W* = 4.74, *p* < 0.001) and a little worried (*W* = 3.95, *p* < 0.05). Participants who reported worrying a lot increased their music listening more than those who reported being not worried (*W* = 4.53, *p* < 0.05). No other between-group significant differences were found. Income change was also a significant predictor of changes to music listening (*χ*^2^ = 17.6, *p* < 0.001, *ε*^2^ = 0.01). Respondents whose income was unaffected reported smaller increases in music listening than those whose income decreased somewhat (*W* = −4.21, *p* < 0.05), increased somewhat (*W* = 4.62, *p* < 0.05), and increased a lot (*W* = 4.01, *p* < 0.05). No other between-group significant differences were found for change in income. Respondents with children under the age of 18 at home increased their music listening more than those who did not (*χ*^2^ = 28.9, *p* < 0.001, *ε*^2^ = 0.01). Respondents who had COVID-19 themselves or knew someone personally who did also increased their music listening more than those who did not (*χ*^2^ = 6.46, *p* < 0.05, *ε*^2^ = 0.01). Living in a region with a high COVID-19 incidence rate, internet type (mobile vs. home internet), and internet speed were not significant predictors of changes to music listening.

#### Personal variables predicting changes to music watching

Results of a Kruskal-Wallis ANOVA showed that age was a significant predictor of changes to music watching (*χ*^2^ = 21, *p* < 0.01, *ε*^2^ = 0.01). Respondents aged 30–44 increased their music watching the most, significantly more than those aged 45–59 (*W* = −4.34, *p* < 0.05) and aged 60 and over (*W* = −7.14, *p* < 0.001). The youngest age group (18–29) increased their music watching more than those aged 60 and over (*W* = −4.22, *p* < 0.05). There were no other significant differences between groups. Pre-pandemic income was also a significant predictor of changes to music watching (*χ*^2^ = 28.95, *p* < 0.001, *ε*^2^ = 0.01). Respondents whose household income was below $35,000 in 2019 reported an overall decrease in music watching; DSCF pairwise comparison indicated this change was significant compared to those who earned $50–75,000 (*W* = 5.31, *p* < 0.01), $75–100,000 (*W* = 5.66, *p* < 0.001), $100–150,000 (*W* = 6.12, *p* < 0.001), and over $150,000 (*W* = 5.09, *p* < 0.01). There were no other significant differences between groups. Members of a visible minority or racialized community increased their music watching more than non-minorities (*χ*^2^ = 4.37, *p* < 0.05, *ε*^2^ = 0.002). Education level was also a significant predictor of changes to music watching (*χ*^2^ = 48.3, *p* < 0.001, *ε*^2^ = 0.02). Respondents with a Bachelor’s degree reported the highest increases in music watching; DSCF pairwise comparison indicated they increased their music watching more than those who did not complete high school (*W* = 4.98, *p* < 0.01), high school graduates (*W* = 8.95, *p* < 0.001), and those with some college or university (*W* = 5.63, *p* < 0.001). Respondents who completed a graduate degree or higher also increased their music watching more than those who did not complete high school (*W* = 3.88, *p* < 0.05) and high school graduates (*W* = 5.07, *p* < 0.01). There were no other significant differences between groups. There were no significant differences for sex.

Changes to music watching were found to differ significantly based on respondents’ feelings about music (*χ*^2^ = 88.50, *p* < 0.001, *ε*^2^ = 0.04). Respondents who reported loving music increased their music watching more than those who like it (*W* = 10.47, *p* < 0.001), were indifferent to it (*W* = 9.01, *p* < 0.001), and dislike it (*W* = 4.72, *p* < 0.01). There were no other significant differences between groups.

Changes to music watching differed significantly for values of Stimulation (*χ*^2^ = 14.2, *p* < 0.05, *ε*^2^ = 0.01), Achievement (*χ*^2^ = 11.9, *p* < 0.05, *ε*^2^ = 0.005), Universalism (*χ*^2^ = 12.58, *p* < 0.05, *ε*^2^ = 0.01), and Tradition (*χ*^2^ = 11.2, *p* < 0.05, *ε*^2^ = 0.005). Pairwise comparisons for Achievement indicated a significant difference between those who responded “A little like me” and “Very much like me” (*W* = −4.5, *p* < 0.05). Pairwise comparisons for Universalism revealed a significant difference between those who responded “Somewhat like me” and “Like me” (*W* = −4.3, *p* < 0.05). There were no significant pairwise comparisons for Stimulation or Tradition.

#### Contextual variables predicting changes to music watching

A Kruskal-Wallis AVOVA for level of worry and changes to music watching revealed significant differences (*χ*^2^ = 43.1, *p* < 0.001, *ε*^2^ = 0.02). Participants who were not worried reported an overall decrease in music watching. This change in music watching was significantly different from those who reported being somewhat worried (*W* = 4.46, *p* < 0.05), worried a lot (*W* = 7.14, *p* < 0.001), and extremely worried (*W* = 5.95, *p* < 0.001). Participants who reported worrying a lot had the highest increase in music watching. They increased their music watching more than those who reported being a little worried (*W* = 6.58, *p* < 0.001) and somewhat worried (*W* = 5.34, *p* < 0.01). Those who reported being extremely worried increased music watching more than those who were a little worried (*W* = 4.66, *p* < 0.01). No other between-group significant differences were found. Income change was also a significant predictor of changes to music watching (*χ*^2^ = 10.93, *p* < 0.001, *ε*^2^ = 0.05, *ε*^2^ = 0.004). Respondents whose income was unaffected reported smaller increases in music watching than those whose income decreased somewhat (*W* = 3.88, *p* < 0.05). No other between-group significant differences were found for change in income. Respondents with children under the age of 18 at home increased their music watching more than those who did not (*χ*^2^ = 21.8, *p* < 0.001, *ε*^2^ = 0.01). Respondents who had COVID-19 themselves or knew someone who did personally also increased their music watching more than those who did not (*χ*^2^ = 12.56, *p* < 0.001, *ε*^2^ = 0.01). Respondents who reported using a mobile provider as their main way of connecting to the internet at home increased their music watching more than those who used a home internet connection (*χ*^2^ = 13.1, *p* < 0.01, *ε*^2^ = 0.01). Internet speed was not a significant predictor of changes to music watching, nor was living in a region with a high COVID-19 incidence rate.

#### Personal variables predicting music discovery

Results of a Kruskal-Wallis ANOVA showed that age was a significant predictor of music discovery (*χ*^2^ = 141.1, *p* < 0.001, *ε*^2^ = 0.06). The youngest age group (18–29) reported the highest level of music discovery, more than those aged 30–44 (*W* = −4.63, *p* < 0.01), 45–59 (*W* = −10.35, *p* < 0.001), and 60 and over (*W* = −15.4, *p* < 0.001). Respondents aged 30–44 reported more music discovery than those aged 45–59 (*W* = −6.36, *p* < 0.001) and aged 60 and over (*W* = −11.94, *p* < 0.001). Respondents aged 45–59 reported more music discovery than those aged 60 and over (*W* = −5.6, *p* < 0.001). Members of a visible minority or racialized community discovered more new music than non-minorities (*χ*^2^ = 22.66, *p* < 0.001, *ε*^2^ = 0.01). Education level was also a significant predictor of music discovery (*χ*^2^ = 11.2, *p* < 0.05, *ε*^2^ = 0.004). Respondents with a Bachelor’s degree discovered more new music than high school graduates (*W* = 4.59, *p* < 0.05). There were no other significant differences between groups. There were no significant differences for sex or pre-pandemic income.

Music discovery was found to differ significantly based on respondents’ feelings about music (*χ*^2^ = 88.50, *p* < 0.001, *ε*^2^ = 0.04). Respondents who reported loving music discovered more music than those who like it (*W* = 18.27, *p* < 0.001), were indifferent to it (*W* = 15.66, *p* < 0.001), and dislike it (*W* = 5.3, *p* < 0.01). Those who like music discovered more than those who were indifferent to it (*W* = 7.17, *p* < 0.001). There were no other significant differences between groups.

Music discovery differed significantly (*p* < 0.05) for each of the 10 PVC values (self-direction, power, stimulation, achievement, tradition, universalism, conformity, security, benevolence, and hedonism). Results are outlined in Table 1. Significant pairwise comparisons are listed in Supplementary Table S6.

#### Contextual variables predicting music discovery

A Kruskal-Wallis AVOVA for level of worry and music discovery revealed significant differences (*χ*^2^ = 31, *p* < 0.001, *ε*^2^ = 0.01). Participants who reported being extremely worried reported the highest music discovery. Their music discovery was significantly different from those who reported being a little worried (*W* = 6.79, *p* < 0.001) and somewhat worried (*W* = 4.72, *p* < 0.01). Those who reported worrying a lot reported more music discovery than those who were a little worried (*W* = 4.72, *p* < 0.001). No other between-group significant differences were found. Income change was also a significant predictor of music discovery (*χ*^2^ = 50.92, *p* < 0.001, *ε*^2^ = 0.05, *ε*^2^ = 0.02). Respondents whose income increased a lot reported the most music discovery. They discovered more music than those whose income was unaffected (*W* = 7.36, *p* < 0.001), decreased somewhat (*W* = 4.68, *p* < 0.01), and decreased a lot (*W* = 3.89, *p* < 0.05). Respondents whose income increased somewhat discovered more new music than those whose income was unaffected (*W* = 7.59, *p* < 0.001). Respondents whose income was unaffected discovered more new music than those whose income decreased somewhat (*W* = −5.65, *p* < 0.001) and decreased a lot (*W* = −4.27, *p* < 0.05). No other between-group significant differences were found for change in income. Respondents with children under the age of 18 at home discovered more music than those without children (*χ*^2^ = 24.6, *p* < 0.001, *ε*^2^ = 0.01). Respondents who had COVID-19 themselves or knew someone who did personally also discovered more music than those who did not (*χ*^2^ = 4.25, *p* < 0.05, *ε*^2^ = 0.002). Respondents who reported using a mobile provider as their main way of connecting to the internet at home discovered more music than those who used a home internet connection (*χ*^2^ = 29.4, *p* < 0.001, *ε*^2^ = 0.01). Internet speed was also a significant predictor of music discovery; those who did not have access to high-speed internet discovered more music than those who did (*χ*^2^ = 10.96, *p* < 0.001, *ε*^2^ = 0.004). Living in a region with a high COVID-19 incidence rate was not a significant predictor of music discovery.

#### Comparison of personal and contextual variables

In comparison of personal and contextual variables to predict music listening for stress relief, music discovery, changes in music listening, and changes in music watching, hierarchical linear regression model 3 accounted for a higher variance of the dependent variable than model 1 and 2 in all four cases. As such, model 3 was selected for further examination. Results are outlined in Table 2. Individual predictors in model 3 [age, sex, feelings about music, PVC values, visible minority status, education level, income level, level of worry, change in income, whether respondents or someone they knew personally caught COVID, having children at home, living in a region of high incidence of COVID-19, having high-speed internet access and type of internet plan] were investigated.

**Table 2:** ANOVA for Hierarchical regression analyses models for music listening for stress relief, music listening, music watching and music discovery.

		Model 1	Model 2	Model 3
		Age, sex, feelings about music, PVC values	Model 1 variables + level of worry, change in income, you or someone you knew caught COVID-19, children at home, living in a region of high incidence of COVID-19	Model 1 + 2 + education level, visible minority status, income level, high speed internet access, type of internet pla
Music listening for stress relief	*R* ^2^	0.20	0.21	0.21
	Adjusted *R*^2^	0.20	0.20	0.20
	df	12	17	21
	*F*	32.91	23.70	19.45
	*p*	<0.001	<0.001	<0.001
Music listening	*R*^2^	0.05	0.06	0.07
	Adjusted *R*^2^	0.04	0.05	0.06
	df	12	17	21
	*F*	6.24	5.93	5.44
	*p*	<0.001	<0.001	<0.001
Music watching	*R*^2^	0.05	0.07	0.08
	Adjusted *R*^2^	0.04	0.06	0.07
	df	12	17	21
	*F*	6.07	6.52	6.71
	*p*	<0.001	<0.001	<0.001
Music discovery	*R*^2^	0.18	0.19	0.19
	Adjusted *R*^2^	0.17	0.18	0.18
	df	12	17	21
	*F*	27.78	20.63	17.05
	*p*	<0.001	<0.001	<0.001

In investigating music listening for stress relief (Table 3), results showed that feelings about music (*β* = 0.39, *t* = 16.76, *p* < 0.01), age (*β* = −0.11, *t* = −4.34, *p* < 0.01), PVC values of universalism (*β* = −0.09, *t* = −2.78, *p* < 0.01) and power (*β* = −0.11, *t* = −2.73, *p* < 0.01) and visible minority status (*β* = 0.05, *t* = 2.07, *p* < 0.05) were significant predictors in the model. That is, liking or loving music and being a member of a visible minority status predicted a higher likelihood of listening to music for stress relief, while an increase in age and a higher score on PVC values of universalism and power predicted a lower likelihood of listening to music for stress relief. No other significant relationships were found.

**Table 3:** Hierarchical linear regression results for music listening for stress relief.

					95%CI	
Model 3	*β*	*t*	S.E.	*p*-value	Lower bound	Upper bound
Constant		−0.70	0.03	0.48	−0.09	0.04
Age	−0.11	−4.35	0.03	**<0.001**	−0.17	−0.06
Sex	−0.00	−0.10	0.02	0.92	−0.05	0.04
Feelings about music	0.39	16.76	0.02	**<0.001**	0.34	0.44
Self-direction	0.01	0.43	0.04	0.67	−0.07	0.11
Benevolence	0.00	0.09	0.04	0.93	−0.08	0.09
Universalism	−0.09	−2.78	0.05	**0.01**	−0.25	−0.04
Hedonism	−0.04	−1.17	0.04	0.24	−0.12	0.03
Security	−0.04	−1.23	0.04	0.22	−0.13	0.03
Conformity	−0.02	−0.81	0.04	0.42	−0.11	0.04
Stimulation	−0.01	0.25	0.04	0.81	−0.07	0.08
Tradition	0.011	0.40	0.03	0.69	−0.05	0.07
Power	−0.11	−2.72	0.03	**0.01**	−0.13	−0.02
Level of worry	0.03	1.30	0.02	0.19	−0.02	0.08
Change in income	−0.02	−1.06	0.02	0.29	−0.07	0.02
You or someone you knew caught COVID-19	−0.01	−0.51	0.02	0.61	−0.06	0.03
Children at home	0.04	1.71	0.02	0.09	−0.01	0.09
Living in a region of high incidence of COVID-19	0.01	0.34	0.02	0.73	−0.04	0.05
Education level	−0.03	−1.17	0.02	0.24	−0.08	0.02
Visible minority status	0.05	2.09	0.02	**0.04**	0.00	0.10
Income level	0.01	0.19	0.02	0.85	−0.05	0.05
High speed internet access	0.00	0.02	0.02	0.99	−0.04	0.04

Excluded variable: achievement. Bold values indicate statistical significance.

In investigating changes in music listening (Table 4), results showed that PVC values of hedonism (*β* = −0.12, *t* = −3.49, *p* < 0.01), universalism (*β* = −0.09, *t* = −2.47, *p* < 0.05) and power (*β* = −0.09, *t* = −2.18, *p* < 0.05), feelings about music (*β* = 0.16, *t* = 6.12, *p* < 0.01), level of worry (*β* = 0.06, *t* = 2.23, *p* < 0.05), having children at home (*β* = 0.09, *t* = 3.52, *p* < 0.01) and visible minority status (*β* = 0.08, *t* = 3.05, *p* < 0.01) were significant predictors in the model. In other words, a lower score on PVC values of hedonism, universalism and power, liking or loving music, a higher level of worry, having children at home during the pandemic and being a member of a visible minority predicted an increase in listening to music during the pandemic. No other significant relationships were found.

**Table 4:** Hierarchical linear regression results for music listening.

					95%CI	
Model 3	*β*	*t*	S.E.	*p*-value	Lower bound	Upper bound
Constant		1.30	0.03	0.20	−0.02	0.11
Age	−0.02	−0.64	0.03	0.52	−0.07	0.04
Sex	−0.01	−0.47	0.02	0.64	−0.06	0.04
Feelings about music	0.16	6.14	0.02	**<0.001**	0.10	0.20
Self-direction	−0.02	−0.66	0.05	0.51	−0.12	0.06
Benevolence	−0.06	−1.76	0.04	0.08	−0.17	0.01
Universalism	−0.09	−2.47	0.06	0.01	−0.25	−0.03
Hedonism	−0.12	−3.49	0.04	**<0.001**	−0.23	−0.06
Security	−0.06	−1.70	0.04	0.09	−0.16	0.01
Conformity	−0.03	−0.88	0.04	0.38	−0.12	0.04
Stimulation	−0.03	−0.70	0.04	0.48	−0.11	0.05
Tradition	−0.05	−1.85	0.03	0.06	−0.12	0.00
Power	−0.09	−2.18	0.03	0.03	−0.12	−0.01
Level of worry	0.06	2.23	0.03	0.03	0.01	0.11
Change in income	0.02	0.67	0.02	0.50	−0.03	0.07
You or someone you knew caught COVID-19	0.02	0.64	0.03	<0.52	−0.03	0.06
Children at home	0.09	3.52	0.02	**<0.001**	0.04	0.14
Living in a region of high incidence of COVID-19	0.01	0.42	0.03	0.68	−0.04	0.06
Education level	−0.01	−0.33	0.03	0.74	−0.06	0.04
Visible minority status	0.08	3.05	0.03	0.00	0.03	0.13
Income level	0.03	1.15	0.02	0.25	−0.02	0.08
High speed internet access	0.03	1.27	0.02	0.20	−0.02	0.08

Excluded variable: achievement. Bold values indicate statistical significance.

In investigating changes in music watching (Table 5), Results showed that feelings about music (*β* = 0.18, *t* = 7.28, *p* < 0.01), level of worry (*β* = 0.06, *t* = 2.30, *p* < 0.05), having children at home (*β* = 0.11, *t* = 4.09, *p* < 0.01) and education level (*β* = 0.11, *t* = 4.22, *p* < 0.01) were significant predictors in the model. In other words, liking or loving music, a higher level of worry, having children at home during the pandemic, and a higher education level predicted an increase in music watching during the pandemic. No other significant relationships were found.

**Table 5:** Hierarchical linear regression results for music watching.

					95%CI	
Model 3	*β*	*t*	S.E.	*p*-value	Lower bound	Upper bound
Constant		0.64	0.03	0.52	−0.05	0.09
Age	−0.02	−0.57	0.03	0.57	−0.07	0.04
Sex	0.01	0.56	0.02	0.58	−0.04	0.06
Feelings about music	0.18	7.21	0.02	**<0.001**	0.13	0.23
Self-direction	0.01	0.22	0.05	0.83	−0.08	0.10
Benevolence	0.03	0.77	0.04	0.44	−0.05	0.12
Universalism	0.04	1.10	0.06	0.27	−0.05	0.18
Hedonism	−0.01	−0.37	0.04	0.71	−0.10	0.07
Security	−0.02	−0.50	0.05	0.62	−0.11	0.07
Conformity	0.00	−0.09	0.04	0.93	−0.09	0.08
Stimulation	0.03	0.63	0.04	0.53	−0.06	0.11
Tradition	0.01	0.26	0.03	0.80	−0.06	0.07
Power	0.04	1.03	0.03	0.30	−0.03	0.09
Level of worry	0.06	2.30	0.03	0.02	0.01	0.11
Change in income	0.03	1.06	0.03	0.29	−0.02	0.08
You or someone you knew caught COVID-19	0.02	0.80	0.02	0.43	−0.03	0.07
Children at home	0.11	4.09	0.03	**<0.001**	0.05	0.16
Living in a region of high incidence of COVID-19	0.01	0.57	0.02	0.57	−0.03	0.06
Education level	0.11	4.22	0.03	**<0.001**	0.06	0.17
Visible minority status	0.02	0.80	0.03	0.43	−0.03	0.07
Income level	0.02	0.69	0.03	0.49	−0.03	0.07
High speed internet access	0.03	1.37	0.02	0.17	−0.01	0.08

Excluded variable: achievement. Bold values indicate statistical significance.

In investigating music discovery (Table 6), Results showed that feelings about music (*β* = 0.31, *t* = 13.21, *p* < 0.01), age (*β* = −0.13, *t* = −5.04, *p* < 0.01), level of worry (*β* = 0.05, *t* = 1.98, *p* < 0.05), having children at home (*β* = 0.06, *t* = 2.57, *p* < 0.05) and the PVC values of hedonism (*β* = −0.11, *t* = −3.39, *p* < 0.01), security (*β* = −0.08, *t* = 2.49, *p* < 0.05), and tradition (*β* = −0.07, *t* = −2.75, *p* < 0.01) were significant predictors in the model. In other words, liking music, a decrease in age, a higher level of worry, having children at home during the pandemic, a lower score on the PVC values of hedonism, security, and tradition predicted an increase in music discovery during the pandemic. No other significant relationships were found.

**Table 6:** Hierarchical linear regression results for music discovery.

					95%CI	
Model 3	*β*	*t*	S.E.	*p*-value	Lower bound	Upper bound
Constant		1.48	0.03	0.14	−0.02	0.11
Age	−0.13	−5.04	0.03	**<0.001**	−0.19	−0.08
Sex	−0.01	−0.40	0.02	0.69	−0.06	0.04
Feelings about music	0.31	13.21	0.02	**<0.001**	0.27	0.36
Self-direction	−0.01	−0.30	0.04	0.76	−0.10	0.07
Benevolence	−0.02	−0.56	0.04	0.58	−0.11	0.06
Universalism	−0.06	−1.75	0.06	0.08	−0.20	0.01
Hedonism	−0.11	−3.39	0.04	**<0.001**	−0.22	−0.06
Security	−0.08	−2.49	0.04	0.01	−0.19	−0.02
Conformity	−0.06	−1.71	0.04	0.09	−0.15	0.01
Stimulation	0.05	1.24	0.04	0.21	−0.03	0.13
Tradition	−0.07	−2.75	0.03	0.01	−0.15	0.03
Power	−0.08	−1.95	0.03	0.05	−0.11	0.00
Level of worry	0.05	1.98	0.02	0.05	0.00	0.10
Change in income	0.04	1.68	0.02	0.09	−0.01	0.09
You or someone you knew caught COVID-19	−0.01	−0.53	0.02	0.60	−0.06	−0.03
Children at home	0.06	2.57	0.02	0.01	0.02	0.11
Living in a region of high incidence of COVID-19	0.01	0.36	0.02	0.72	−0.04	0.06
Education level	−0.03	1.28	0.03	0.20	−0.02	0.08
Visible minority status	0.04	1.83	0.02	0.07	0.00	0.09
Income level	−0.03	−0.99	0.03	0.32	−0.08	0.03
High speed internet access	0.02	0.97	0.02	0.33	−0.02	0.07

Excluded variable: achievement. Bold values indicate statistical significance.

### Summary of results

Analyses were conducted to examine the individual and contextual predictors of music listening for stress relief, changes in music listening, changes in music watching, and music discovery during the COVID-19 pandemic. Overall, the results support previous findings that suggest that the COVID-19 pandemic has had a significant impact on music-related behaviors, with individual and contextual factors predicting music listening for stress relief, changes in music listening, changes in music watching, and music discovery. Across all four behaviors, liking or loving music was a significant predictor. A higher level of worry and having children at home during the pandemic were predictors of increased music listening, music watching and music discovery. Being a member of a visible minority group predicted increased listening to music for stress relief and changes in music listening. In terms of specific predictors for each behavior the findings indicated that the PVC values of hedonism, power, universalism, security, and tradition had varying effects on the likelihood of engaging in music-related behaviors.

## Discussion

To our knowledge, this study was the first to examine the relationship between personal values and music consumption during the pandemic. While we found a relation between each of the 10 PVC values and music listening for stress relief, only universalism (understanding, appreciating, and protecting the welfare of all) and power (placing importance on social status, prestige, and dominance over people and resources) were found to be significant negative predictors in the hierarchical linear regression model, with higher scores on each predicting lower levels of music listening for stress relief. Universalism falls under the higher-order dimension of self-transcendence, while power falls under self-enhancement (Schwartz and Bardi, 2001).

The relationship between personal values and stress-coping behaviors, such as listening to music, is complex and can be influenced by many factors, including individual differences, cultural norms, and situational factors (Boniwell and Zimbardo, 2004). One possible explanation for the finding that higher levels of universalism and power predicted lower levels of listening to music for stress relief is that individuals who prioritize these values may use different strategies for managing stress. For example, people who prioritize universalism may be more likely to seek social support or engage in altruistic behaviors as a way of coping with stress (Schwartz, 1992). Similarly, individuals who place a high value on power may be more likely to use problem-solving or action-oriented coping strategies, such as exercise or goal-setting, to manage stress (Bardi and Schwartz, 2003). Openness to change (placing high importance on the values of stimulation, self-direction, and hedonism) was previously shown to correlate positively with using music for emotional purposes (Manolika et al., 2021). This effect was not observed in our sample. Further research is needed to examine how PVC values relate to specific uses of music and how these relationships vary across individuals, cultures, and contexts.

Our findings suggest personal values also play a role in changes in music listening during the pandemic. Specifically, the Kruskal-Wallis ANOVAs revealed that achievement and stimulation values were significantly related to changes in music listening; individuals who place lower importance on achieving success and experiencing excitement showed greater increases in their music listening. However, the regression models revealed that hedonism and power were significant predictors of changes in music listening. One possible explanation for these discrepancies is that achievement and stimulation are related to changes in music listening indirectly, through their association with hedonism and power values. For example, individuals who are less driven by stimulation may also be less likely to seek out certain experiences for pleasure (hedonism). Both stimulation and hedonism map onto the higher order value of Openness to Change. Individuals who place lower priority on achievement may be less likely to want to exert control (power); both achievement and power map onto the higher order value of Self-Enhancement. While further study is needed to understand the role of personal values in music listening habits, they seem to be important predictors of changes in music listening during the pandemic. The effects of personal values are complex and may be interrelated.

Lower scores on the PVC value of hedonism predicted both a greater increase in music listening and higher levels of music discovery. Hedonism refers to the pursuit of pleasure and enjoyment. These findings suggest that individuals who place lower importance on seeking pleasure demonstrate greater increases in music listening and higher levels of music discovery. That those who place less importance on hedonism (i.e., pleasure and self-gratification) showed greater increases in music listening and discovered more music might suggest they were seeking musical experiences for reasons other than pleasure such as social connection or emotional coping. Hedonism shares elements of both openness to change and self-enhancement making its relationship to value dimensions complex (Schwartz, 2012).

Placing less importance on both power and universalism also predicted a greater increase in listening to music. The power value refers to the desire to control or dominate others and to attain social status and prestige, while universalism refers to understanding, appreciating, and protecting others. That individuals who score low on power and universalism show greater increases in listening to music may suggest the behavior is not related to a desire to either control or protect others. Instead, it may indicate that increases in music listening are more driven by factors related to the self, such as personal enjoyment or relaxation. Individuals who score lower on power and universalism may be less concerned with others and more focused on their own interests and hobbies. Listening to music may be a personal interest that is valued for its intrinsic enjoyment, rather than for its ability to increase one’s social status or offer support to others.

Lower scores on security and tradition predicted more music discovery during the pandemic. Security and tradition map onto the higher-order value of conservation, the opposite of openness to change. Conservation values emphasize preserving traditional social norms and values, maintaining order and stability, and protecting the social structure from change. Resistance to change and preservation of the past might explain why individuals with lower scores these values would be more likely to discover music during the pandemic. In contrast, openness to change values (including hedonism) prioritize innovation, change, and a willingness to challenge traditional values. That music discovery was predicted by both low levels of conservation and hedonism suggests the behavior is complex, multi-faceted, and has elements that could be both traditional and innovative.

While changes to music watching were significantly related to values of stimulation, achievement, universalism, and power, none of the PVC values predicted changes to music watching behaviors in the regression models. One possible explanation for these discrepancies is that Kruskal-Wallis ANOVAs and regression models are sensitive to different aspects of the data. Kruskal-Wallis ANOVAs are non-parametric tests that compare the medians of different groups, whereas regression models assume a linear relationship between the dependent and independent variables. It is possible the relation between changes to music watching and each value (stimulation, achievement, universalism, and power) is non-linear, resulting in the Kruskal-Wallis ANOVAs detecting significant effects while the regression models did not. Further study is warranted to explore the relations between PVCs and changes to music watching behavior.

Another objective of this paper was to examine the role of personal (demographic) variables and contextual variables in music consumption during the first COVID-19 lockdown in Canada. We found significant effects of age, sex, minority status, and feelings about music on music listening for stress relief. Women were significantly more likely than men to listen to music to relieve stress. Individuals from the youngest age group (18–29 years) were the most likely to use music for stress relief. Respondents who indicated they were members of a visible minority or racialized community were more likely to listen to music to relieve stress than non-minorities. These findings can be interpreted based on reports that women, younger individuals, and racial minorities suffered higher emotional impacts than men, older adults, and non-minorities (Canadian Association of Mental Health, 2020). Women and individuals aged 18–24 reported a greater decline in mental health than men and those aged 75 or older. Racial minorities were more likely to have trouble coping and worried more about being safe from physical or emotional domestic violence. However, the observed effects persisted even after we accounted for the level of worry in our analysis. One explanation could be that the questionnaire did not measure stress directly and assessed worry using only one question, which might not have adequately captured the emotional impacts of the pandemic.

The effect sizes for sex, age, and minority differences in music listening for stress relief were small, so these findings must be interpreted with caution. Sex was not found to be a significant predictor of music listening for stress relief in the hierarchical linear regression model; age, minority status, and feelings about music were. These findings support and extend previous reports. While women seem to be more likely than men to listen to music to reduce negative emotions and alleviate loneliness in general (North, 2010; Lonsdale and North, 2011; Chamorro-Premuzic et al., 2012; Ter Bogt et al., 2017), some studies have found no significant effect of gender on music’s perceived efficacy for well-being during the pandemic (Fink et al., 2021; Martínez-Castilla et al., 2021). Younger individuals are more likely to listen to music for socio-emotional purposes both in general and more specifically in the context of COVID-19 (Lonsdale and North, 2011; Martín et al., 2021; Martínez-Castilla et al., 2021). Previous studies on music and well-being during the pandemic have not reported on minority status. Our findings that individuals who reported being a member of a visible minority or racialized community adds to the literature.

In our investigation, individuals who reported loving or liking music were significantly more likely to listen to music for stress relief than those who reported an indifferent or unfavorable attitude towards music. However, individuals who report a more positive attitude towards music may simply be more likely to listen to music for any reason. In our analyses, those who reported loving or liking music did increase their music listening more than those who reported being indifferent or disliking or hating music. We are unsure from our data if the observed effect is limited to listening to music for stress relief or if it also applies to other uses of music, such as listening to music for entertainment or social purposes. In another study, changes in overall music listening behavior were associated with listening to music specifically for stress relief (Fink et al., 2021), but further research is warranted to investigate feelings about music and music listening for purposes beyond stress relief both in general and in specific contexts such as lockdown. Importance of music has been associated with more extensive use of music listening as a coping strategy (Ribeiro et al., 2021) and to promote well-being (Martínez-Castilla et al., 2021) during the pandemic.

While higher levels of worry, having children at home, and change to income corresponded with listening to music for stress relief, none of the contextual variables investigated were significant predictors in the hierarchical linear regression model. This suggests that the emotional impact of situational aspects, rather than the situations themselves, may be more relevant for changes in musical engagement. A comparison of the predictive power of personal and contextual variables indicated personal variables accounted for a larger variance in music listening for stress relief than contextual variables. This supports previous research on the importance of personal variables in predicting use of music for emotional reasons (Martínez-Castilla et al., 2021).

Personal and contextual variables also significantly predicted changes to both music listening and music watching, as well as music discovery in our sample. Feelings about music and having children under 18 at home significantly predicted all three behaviors. Though not a predictor in the hierarchal linear model, one particularly interesting finding was that respondents who reported using a mobile provider as their main way of connecting to the internet at home and those who did not have access to high-speed internet discovered more music. It may be that internet availability and speed limited access to other forms of entertainment, such as video streaming and online gaming, resulting in more active engagement with music. Based on our data, one could argue that while music engagement is impacted by both the context and the person, the use of music for emotional purposes is impacted more heavily by personal variables, including values.

While this paper offers meaningful results and discussion, we faced several limitations. First, many questions in the survey required the participants to compare their current behavior with their behavior before the pandemic. Given the retrospective nature of these questions, there is potential bias in respondents’ answers. Studies employing the experience sampling method (ESM), assessing current subjective experience during natural everyday music episodes, could yield different results. Previous studies using this method suggest individual differences are of minor importance, while situational variables (e.g., listening alone vs. with others, time of the day, self-chosen music, and initial mood state) predict both function of music listening and music selection (Randall and Rickard, 2017; Greb et al., 2019). Thus, results should be interpreted with caution. Future studies employing ESM during times of crises such as the pandemic are warranted. Having pre-crisis baseline assessments would provide a more comprehensive understanding of changes in behavior and mood during times of crisis. Additionally, the cross-sectional and correlational nature of the study means that conclusions about causality between the variables cannot be drawn. Finally, we used an existing dataset that was designed by Abacus Canada for Music Canada for market and industry research purposes. We did not control the items included or the wording and the chronological order of the questions in the survey.

Overall, this paper highlights the importance of considering individual and contextual factors when examining music listening behavior during the pandemic. The results support that music may serve as a coping mechanism for individuals experiencing high levels of stress, and that personal values and socio-economic factors may impact the extent to which music is used for this purpose. Furthermore, the findings suggest that changes in music consumption may be related to the ways in which individuals adapt to the pandemic, such as by spending more time at home with family. By shedding light on the factors that predict music listening behavior during the pandemic, these results may help inform interventions aimed at promoting mental health and well-being during times of crisis.

## Data availability statement

The datasets presented in this study can be found in online repositories. The names of the repository/repositories and accession number(s) can be found in the article/Supplementary material.

## Ethics statement

The studies involving human participants were reviewed and approved by McGill University Research Ethics Board. Written informed consent for participation was not required for this study in accordance with the national legislation and the institutional requirements.

## Author contributions

A version of this report was submitted in partial fulfillment for the B.A. honors psychology degree at McGill University by YD, under the supervision of DL. YD, LF, and DL contributed equally to the conceptualization, design, methods, and writing the manuscript. YD and LF performed the statistical analysis. All authors contributed to the article and approved the submitted version.

## Funding

This project was funded in part by the Social Sciences and Humanities Research Council of Canada (grant #: 1008–2020-0038) and the Natural Sciences and Engineering Research Council of Canada (grant #: RGPIN-2016-05407) awarded to DL.

## Conflict of interest

The authors declare that the research was conducted in the absence of any commercial or financial relationships that could be construed as a potential conflict of interest.

## Publisher’s note

All claims expressed in this article are solely those of the authors and do not necessarily represent those of their affiliated organizations, or those of the publisher, the editors and the reviewers. Any product that may be evaluated in this article, or claim that may be made by its manufacturer, is not guaranteed or endorsed by the publisher.
