# Supplementary material for: Personal and contextual variables predict music consumption during the first COVID-19 lockdown in Canada
Source: Front Psychol. 2023 Jun 14;14:1116857. doi: 10.3389/fpsyg.2023.1116857 (PMC10300573; doi:10.3389/fpsyg.2023.1116857)
Supplement: Supplementary file 2 [file Data_Sheet_1.docx]

**Table S1 | Descriptive characteristics of the sample**

| Variable | *n* | | | % | |  |  |
| --- | --- | --- | --- | --- | --- | --- | --- |
| Sex (Questionnaire provided to us did not ask about Gender) | | | | | |  |  |
| Male | 1143 | | | 45.7 | |  |  |
| Female | 1357 | | | 54.3 | |  |  |
| Age | | | | | |  |  |
| 18 ­- 29 | 482 | | | 19.3 | |  |  |
| 30 - 44 | 677 | | | 27.1 | |  |  |
| 45 - 59 | 689 | | | 27.6 | |  |  |
| 60 and over | 652 | | | 26.1 | |  |  |
| Feelings about music |  | | |  | |  |  |
| Love it | 1405 | | | 56.2 | |  |  |
| Like it | 891 | | | 35.6 | |  |  |
| Indifferent about it | 186 | | | 7.4 | |  |  |
| Dislike it | 11 | | | 0.4 | |  |  |
| Hate it | 7 | | | 0.3 | |  |  |
| Minority status: Member of a visible minority/racialized community | | | | | |  |  |
| Yes | 403 | | | 83.9 | |  |  |
| No | 2097 | | | 16.1 | |  |  |
| Education level | | | | |  | |  |
| No High School | | 56 | 2.2 | |  |  |  |
| High School | | 594 | 23.8 | |  |  |  |
| College or some university | | 895 | 35.8 | |  |  |  |
| Bachelor’s Degree | | 668 | 26.7 | |  |  |  |
| Post-Graduate Work or Higher | | 287 | 11.5 | |  |  |  |
| Income | | | | |  |  |  |
| Less than $35,000 | | 522 | 20.9 | |  |  |  |
| $35,001 to $50,000 | | 356 | 14.2 | |  |  |  |
| $50,001 to $75,000 | | 491 | 19.6 | |  |  |  |
| $75,001 to $100,000 | | 408 | 16.3 | |  |  |  |
| $100,001 to $150,000 | | 362 | 14.5 | |  |  |  |
| Over $150,000 | | 154 | 6.2 | |  |  |  |
| Rather not say | | 207 | 8.3 | |  |  |  |

**Table S2 | Table S2 Values (PVQ) and Descriptions (Schwartz, 2012)**

| **Value** | **Description** |
| --- | --- |
| Power | Social status, prestige, dominance over people and resources |
| Achievement | Personal success through demonstrated competence |
| Hedonism | Pleasure or gratification for self. |
| Stimulation | Excitement, novelty, and challenge |
| Self-direction | Independence in thought and action, exploration |
| Universalism | Understanding, appreciation, and protection for the welfare of all |
| Benevolence | Enhancing welfare of those with whom one is closely affiliated (friends, family) |
| Conformity | Obedience, restraint of actions and impulses that could violate social norms or harm others |
| Security | Safety, stability of society, relationships and self. |
| Tradition | Respect, commitment, and acceptance of customs of one’s culture or religion |
| **Higher order value** | **Description** |
| Self-Enhancement | (Comprises power and achievement) These values focus on self-interest, success and dominance over others |
| Openness to Change | (Comprises self-direction, stimulation, hedonism) These values focus on autonomy in thought, action, feelings and readiness for change |
| Transcendence | (Comprises universalism, benevolence) These values focus on the welfare and interests of others |
| Conservation | (Comprises security, conformity, tradition) These values focus on order, self-restraint, preservation of past and resistance to change |

**Table S3 | Contextual Variables Responses**

| Variable | *n* | % |  |  |
| --- | --- | --- | --- | --- |
| Level of worry | | |  |  |
| Not worried at all | 135 | 5.4 |  |  |
| A little worried | 631 | 25.2 |  |  |
| Somewhat worried | 738 | 29.5 |  |  |
| Worried a lot | 596 | 23.8 |  |  |
| Extremely worried | 400 | 16 |  |  |
| Change to income | | |  |  |
| Decreased a lot | 332 | 13.3 |  |  |
| Decreased somewhat | 772 | 30.9 |  |  |
| Been unaffected | 946 | 37.8 |  |  |
| Increased somewhat | 309 | 12.4 |  |  |
| Increased a lot | 141 | 5.6 |  |  |
| Children under 18 at home | | |  |  |
| Yes | 1939 | 77.6 |  |  |
| No | 561 | 22.4 |  |  |
| Living in a region of high incidence of COVID-19 | | |  |  |
| High* | 932 | 37.3 |  |  |
| Low | 1138 | 45.52 |  |  |
| Do you have high-speed internet access | | |  |  |
| Yes | 1939 | 77.6 |  |  |
| No | 121 | 4.8 |  |  |
| No response | 440 | 17.6 |  |  |
| Type of internet plan | | |  |  |
| Home internet connection (Wired or Wi-Fi) | 2060 | 82.4 |  |  |
| Wireless Plan | 422 | 16.9 |  |  |
| No internet access | 18 | 0.7 |  |  |
| You or someone close to you getting COVID-19 | | |  |  |
| No, neither | 2129 | 85.2 |  |  |
| Yes, me | 23 | 0.9 |  |  |
| Yes, someone I know | 354 | 14.2 |  |  |

*Regions include: Island of Montreal and Laval (Quebec), Region around Montreal (Quebec), Eastern [postal code starts with K] (Ontario), Toronto [postal code starts with M] (Ontario), GTA [postal code starts with L] (Ontario)

**Table S4 | Dependent Variables Responses**

| Listening to music for stress relief | | |
| --- | --- | --- |
| Strongly agree | 615 | 24.6 |
| Agree | 1356 | 54.2 |
| Disagree | 406 | 16.2 |
| Strongly Disagree | 123 | 4.9 |
| Change in music listening | | |
| Much more than usual | 409 | 16.4 |
| More than usual | 473 | 18.9 |
| About the same as usual | 1365 | 54.6 |
| Less than usual | 180 | 7.2 |
| Much less than usual | 73 | 2.9 |
| Change in music watching: Watching music videos | | |
| Much more than usual | 309 | 12.4 |
| More than usual | 452 | 18.1 |
| About the same as usual | 1381 | 55.2 |
| Less than usual | 177 | 7.1 |
| Much less than usual | 181 | 7.2 |
| Change in music watching: Watching recorded live concerts | | |
| Much more than usual | 225 | 9.0 |
| More than usual | 382 | 15.3 |
| About the same as usual | 1342 | 53.7 |
| Less than usual | 224 | 9.0 |
| Much less than usual | 327 | 13.1 |
| Change in music watching: Watching online video content from musicians | | |
| Much more than usual | 315 | 12.6 |
| More than usual | 509 | 20.4 |
| About the same as usual | 1251 | 50.0 |
| Less than usual | 193 | 7.7 |
| Much less than usual | 232 | 9.3 |
| Music discovery | | |
| Music discovery: Discovering new musicians and artists | | |
| Strongly agree | 226 | 9.0 |
| Agree | 862 | 34.5 |
| Disagree | 1027 | 41.1 |
| Strongly disagree | 385 | 15.4 |
| Music discovery: Finding new content about music and musicians I love | | |
| Strongly agree | 254 | 10.2 |
| Agree | 1155 | 46.2 |
| Disagree | 818 | 32.7 |
| Strongly disagree | 273 | 10.9 |

**Table S5.** Significant DSCF pairwise comparisons among PVC values for music for stress relief.

|  | | | W | p |
| --- | --- | --- | --- | --- |
|  | A little like me | Not at all like me | 10.86 | < .001 |
|  | A little like me | Not like me | 4.10 | 0.04 |
| Self direction | A little like me Like me  Not at all like me | Somewhat like me Not at all like me  Not like me | -4.41  6.31  -8.15 | 0.02  < .001  < .001 |
|  | Not at all like me | Somewhat like me | -12.46 | < .001 |
|  | Not like me | Somewhat like me | -7.67 | < .001 |
|  | A little like me | Not at all like me | 12.43 | < .001 |
|  | A little like me | Not like me | 5.39 | 0.002 |
|  | Like me | Not at all like me | 5.34 | 0.002 |
| Benevolence | Not at all like me | Not like me | -9.35 | < .001 |
|  | Not at all like me | Somewhat like me | -9.81 | < .001 |
|  | Not at all like me | Very much like me | -4.53 | 0.02 |
|  | Not like me | Somewhat like me | -4.65 | 0.01 |
|  | A little like me | Not at all like me | 8.34 | < .001 |
|  | Like me | Not at all like me | 4.20 | 0.04 |
| Universalism | Not at all like me | Not like me | -7.96 | < .001 |
|  | Not at all like me | Somewhat like me | -9.03 | < .001 |
|  | Not like me | Somewhat like me | -4.52 | 0.02 |
|  | A little like me | Not at all like me | 11.79 | < .001 |
|  | A little like me | Not like me | 5.64 | < .001 |
| Hedonism | Like me  Not at all like me | Not at all like me  Not like me | 8.51  -8.13 | < .001  < .001 |
|  | Not at all like me | Somewhat like me | -12.10 | < .001 |
|  | Not like me | Somewhat like me | -7.22 | < .001 |
|  | A little like me | Not at all like me | 10.01 | < .001 |
| Security | Like me  Not at all like me | Not at all like me  Not like me | 6.16  -8.97 | < .001  < .001 |
|  | Not at all like me | Somewhat like me | -7.90 | < .001 |
|  | A little like me | Not at all like me | 8.02 | < .001 |
|  | A little like me | Not like me | 5.40 | 0.002 |
|  | Like me | Not at all like me | 8.97 | < .001 |
| Achievement | Like me  Not at all like me | Not like me  Not like me | 7.29  -4.18 | < .001  0.04 |
|  | Not at all like me | Somewhat like me | -8.71 | < .001 |
|  | Not at all like me | Very much like me | -7.04 | < .001 |
|  | Not like me | Somewhat like me | -6.90 | < .001 |

|  | Not like me | Very much like me | -5.46 | 0.002 |
| --- | --- | --- | --- | --- |
| Conformity | A little like me  Not at all like me Not at all like me | Not at all like me  Not like me Somewhat like me | 6.79  -4.26  -5.12 | < .001 0.03  0.004 |
|  | A little like me | Not at all like me | 12.06 | < .001 |
|  | A little like me | Not like me | 7.96 | < .001 |
|  | Like me | Not at all like me | 10.66 | < .001 |
|  | Like me | Not like me | 7.41 | < .001 |
| Stimulation | Not at all like me | Not like me | -6.38 | < .001 |
|  | Not at all like me | Somewhat like me | -11.15 | < .001 |
|  | Not at all like me | Very much like me | -8.39 | < .001 |
|  | Not like me | Somewhat like me | -6.75 | < .001 |
|  | Not like me | Very much like me | -5.98 | < .001 |
|  | A little like me | Not at all like me | 9.93 | < .001 |
|  | Like me | Not at all like me | 4.20 | 0.04 |
| Tradition | Not at all like me | Not like me | -9.18 | < .001 |
|  | Not at all like me | Somewhat like me | -8.54 | < .001 |
|  | Not at all like me | Very much like me | -4.88 | 0.01 |
|  | A little like me | Somewhat like me | -4.53 | 0.02 |
| Power | Like me  Not at all like me | Not at all like me  Somewhat like me | 5.17  -6.29 | 0.004  < .001 |
|  | Not like me | Somewhat like me | -4.27 | 0.03 |

**Table S6.** Significant DSCF pairwise comparisons among PVC values for music discovery.

|  | | | W | p |
| --- | --- | --- | --- | --- |
|  | A little like me | Not at all like me | 9.62 | < .001 |
| Self direction | Not at all like me  Not at all like me | Not like me  Somewhat like me | -7.46  -9.87 | < .001  < .001 |
|  | Not like me | Somewhat like me | -5.40 | 0.002 |
|  | A little like me | Like me | 4.282 | 0.03 |
| Benevolence | A little like me  Not at all like me | Not at all like me  Not like me | 6.969  -5.769 | < .001  < .001 |
|  | Not at all like me | Somewhat like me | -4.443 | 0.02 |
| Universalism | A little like me | Not at all like me | 6.13 | < .001 |
|  | Not at all like me | Not like me | -5.42 | 0.002 |
|  | A little like me | Not at all like me | 8.54 | < .001 |
| Hedonism | Like me  Not at all like me | Not at all like me  Not like me | 5.01  -6.76 | 0.005  < .001 |
|  | Not at all like me | Somewhat like me | -7.35 | < .001 |
| Security | A little like me | Not at all like me | 4.11 | 0.04 |
|  | A little like me | Not at all like me | 7.55 | < .001 |
|  | A little like me | Not like me | 4.23 | 0.03 |
|  | A little like me | Very much like me | -7.12 | < .001 |
|  | Like me | Not at all like me | 8.77 | < .001 |
|  | Like me | Not like me | 6.76 | < .001 |
| Achievement | Not at all like me | Not like me | -4.57 | 0.02 |
|  | Not at all like me | Somewhat like me | -8.93 | < .001 |
|  | Not at all like me | Very much like me | -9.99 | < .001 |
|  | Not like me | Somewhat like me | -6.68 | < .001 |
|  | Not like me | Very much like me | -8.86 | < .001 |
|  | Somewhat like me | Very much like me | -5.34 | 0.002 |
|  | A little like me | Not at all like me | 11.43 | < .001 |
|  | A little like me | Not like me | 7.57 | < .001 |
|  | A little like me | Very much like me | -7.62 | < .001 |
|  | Like me | Not at all like me | 10.61 | < .001 |
|  | Like me | Not like me | 7.89 | < .001 |
| Stimulation | Like me | Very much like me | -4.92 | 0.007 |
|  | Not at all like me | Not like me | -6.48 | < .001 |
|  | Not at all like me | Somewhat like me | -10.69 | < .001 |
|  | Not at all like me | Very much like me | -11.32 | < .001 |
|  | Not like me | Somewhat like me | -6.97 | < .001 |
|  | Not like me | Very much like me | -10.28 | < .001 |

|  | Somewhat like me | Very much like me | -7.15 | < .001 |
| --- | --- | --- | --- | --- |
| Tradition | A little like me | Not at all like me | 4.27 | 0.03 |
|  | A little like me | Not at all like me | 4.08 | 0.045 |
|  | A little like me | Very much like me | -5.08 | 0.004 |
| Power | Like me  Not at all like me | Not at all like me  Somewhat like me | 5.52  -5.77 | 0.001  < .001 |
|  | Not at all like me | Very much like me | -6.67 | < .001 |
|  | Not like me | Very much like me | -4.97 | 0.006 |
